# High resolution crystal structure of the FAK FERM domain reveals new insights on the Druggability of tyrosine 397 and the Src SH3 binding site

**DOI:** 10.1186/s12860-019-0193-4

**Published:** 2019-05-20

**Authors:** Timothy Marlowe, Alexey Dementiev, Sheila Figel, Andrew Rivera, Michael Flavin, William Cance

**Affiliations:** 1 Interdisciplinary Oncology, University of Arizona College of Medicine - Phoenix, 475 N 5th Street, Phoenix, AZ 85004 USA; 2 Pharmacology and Toxicology, University of Arizona College of Pharmacy, P.O. Box 210207, Tucson, AZ 85721 USA; 3 Shamrock Structures, LLC, 1440 Davey Rd, Woodridge, IL 60517 USA; 4 Structural Biology Center, Argonne National Laboratory, 9700 South Cass Avenue, Argonne, IL 60439 USA; 5 Neuro Oncology, Roswell Park Comprehensive Cancer Center, Elm & Carlton Streets, Buffalo, NY 14263 USA; 6 Cancer Center Division, University of Arizona Cancer Center, 625 N 6th Street, Phoenix, AZ 85004 USA

**Keywords:** Focal adhesion kinase, FERM domain, Drug discovery

## Abstract

**Background:**

Focal Adhesion Kinase (FAK) is a major cancer drug target that is involved in numerous aspects of tumor progression and survival. While multiple research groups have developed ATP-competitive small molecule inhibitors that target the kinase enzyme, recent attention has been focused on the FAK FERM (Band 4.1, Ezrin, Radixin, Moesin) domain that contains key residue Y397 and contributes to many protein-protein interactions. Previous x-ray crystal structures of the FAK FERM domain gave conflicting results on the structure of the Y397 region and therefore the overall druggability.

**Results:**

Here, we report the identification of a higher resolution crystal structure of the avian FAK FERM domain that shows conformational differences in Y397 and surrounding residues in the F1 lobe. In addition, we resolve the residues of the Src SH3 binding site, an area of the FERM domain that has previously shown limited electron density.

**Conclusions:**

These crystallographic data suggest that the Y397 region is highly dynamic and question the druggability of a putative pocket on the F1 lobe. In addition, new electron density data around the Src SH3 binding site provide structural insight on the FAK-Src activation cascade through a putative auto-inhibitory conformation.

## Background

Focal Adhesion Kinase (FAK) is a 125 kDa non-receptor tyrosine kinase that has multiple implications in cancer cell signaling, invasion, proliferation, and metastasis [1, 2]. In addition, FAK is overexpressed in many invasive solid tumors while minimally expressed in normal tissue, making it an attractive therapeutic target for cancer treatment [3–5]. Activation of FAK occurs not only through autophosphorylation of key residue tyrosine 397 (Y397) by FAK itself via the kinase enzyme [6], but also through transphosphorylation by receptor tyrosine kinases which can bind to the FAK N-terminal FERM domain directly [7, 8]. The proto-oncogene Src directly binds to FAK through its SH2 and SH3 domains at phosphorylated FAK Y397 and a proline-rich region in the FERM-Kinase linker, respectively [9, 10]. Src-FAK binding promotes FAK phosphorylation at Y576, Y577, Y861, Y925, and thus the full activation of FAK oncogenic signaling [11, 12]. FAK also functions as an intracellular scaffolding protein through its FERM domain and C-terminal FAT domain regions where FAK forms multiple protein-protein interactions to connect oncogenic signaling pathways [13, 14]. Many efforts have been made to therapeutically target FAK at the catalytic ATP-binding region of the protein (kinase enzyme) and several candidates have entered clinical trials for the treatment of various solid tumors [15–18]. Despite the high-potency of these kinase inhibitors in vitro, results from clinical trials have been disappointing due to multiple factors such as lack of a predictive biomarker, compensatory resistance mechanisms to kinase inhibitors, and lack of targeting key FAK scaffolding regions [19, 20].

As an alternative approach to target FAK, several groups have attempted targeting the FAK FERM domain at a binding site proximal to key residue Y397 as a means to target the FAK activation site directly and inhibit compensatory FAK phosphorylation via transphosphorylation [21–24]. However, significant challenges remain due to the low druggability of the FAK FERM domain and the dynamic nature of the Y397 FERM-Kinase linker region. Previous x-ray crystal structures of the avian FERM domain (2AL6, 2.35 Å) [25] and the human FERM domain (4NY0, 2.8 Å) [26] yielded conflicting results regarding the electron density of residues surrounding Y397. In structure 2AL6, the segment from residue 394–403 can be detected bound to the F1 lobe in a proposed “autoinhibited” state, however in structure 4NY0 there is no clear density of segment 394–403. Structure 2AL6 contains adequate electron density to position 394–403 proximal to the F1 lobe. In addition, structure 2AL6 shows the appearance of a putative druggable pocket proximal to Y397 that has been exploited in multiple structure-based virtual screening campaigns, however this pocket is not visible in structure 4NY0. Nonetheless, it is still unclear whether the region surrounding FAK Y397 can be targeted with small molecules and whether FAK FERM x-ray crystal information can be reliably utilized to guide computer-aided drug design efforts.

Here, we report an x-ray crystal structure of the avian FAK FERM domain at a higher resolution (1.97 Å) than previously reported [25], elucidating new structural features of the FERM-Kinase linker segment that impact the druggability of the Y397 region. In addition, we identify a novel conformation of the Src SH3 binding site (^363^QKEGERALPSIPK^375^) that may have implications in FAK activation via Src. Finally, our crystal structure confirms a previous report identifying the site of dimerization (W266) between two FERM monomers [26].

## Results

### Overall structure

**Fig. 1:**
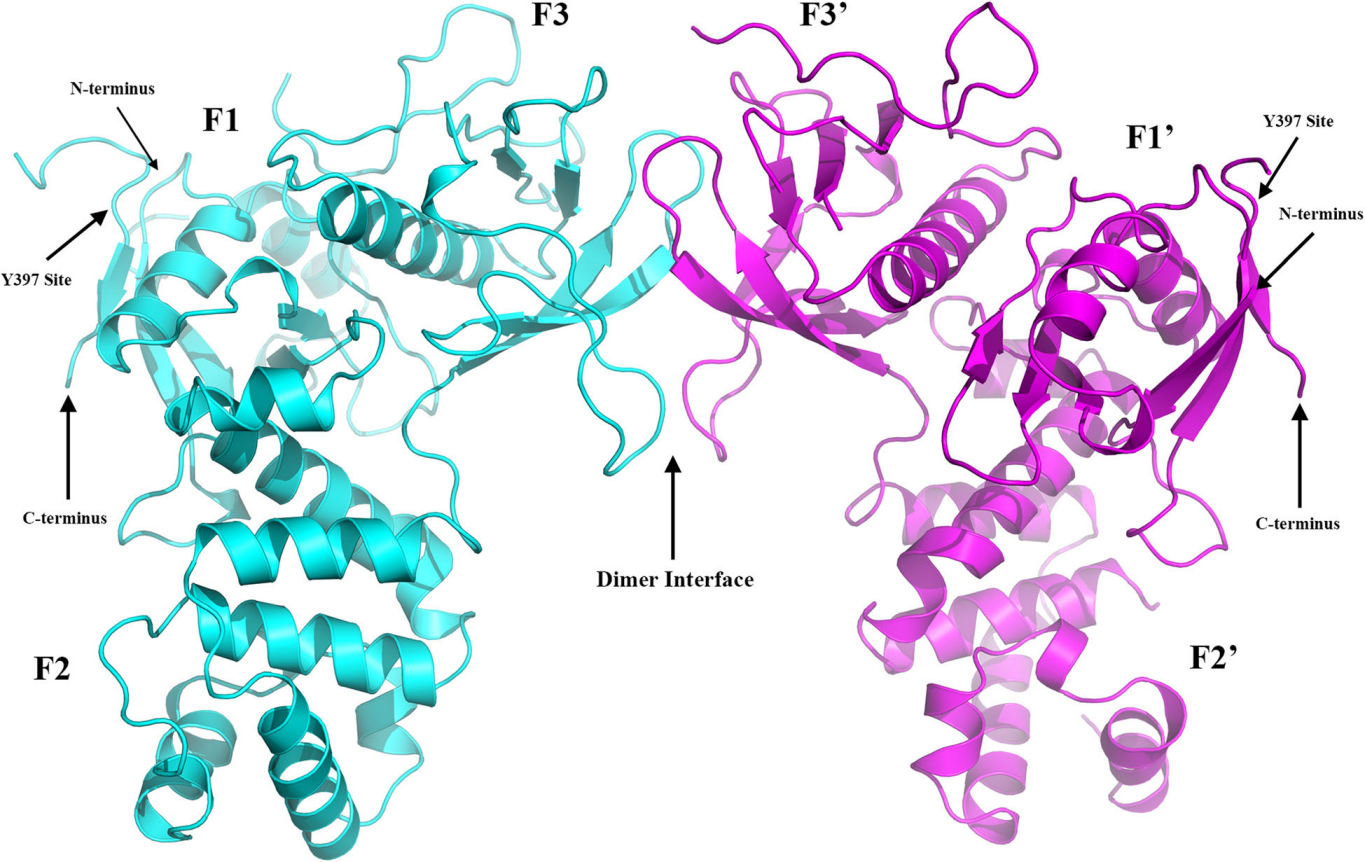
FAK FERM crystal structure. Structural overview of the FAK FERM dimer structure. Monomer A is colored in cyan while monomer B is colored in magenta. N- and C-termini for both monomers are indicated, and three subdomains are labeled as F1, F2, and F3 for molecule A and F1’, F2’, and F3’ for symmetry related molecule B. The key Y397 site for both monomers is indicated, as well as the dimer interface between the two FERM molecules

The crystal structure of the N-terminal domain (residues 31–405, FERM domain) of the avian FAK was solved to a resolution of 1.97 Å by molecular replacement method, using the structure of the FAK FERM (PDB code 2AL6) as search model (Table 1). The protein crystallized in the orthorhombic space group P 212121 with two molecules per asymmetric unit (Fig. 1). The final structure, refined with R_work_ of 17.3 and R_free_ of 21.2, is more ordered than that of the search model [27]. Almost all residues are ordered in the electron density map except residues 376–390 and 403–405 in molecule A and residues 31–33, 182–187, 311–313, 377–393, and 403–405 in molecule B. The previous crystal structure of the FAK FERM domain (4NY0) determined at 2.8 Å resolution did not have adequate electron density for the critical Src SH3 binding motif (residues 363–375) to be visible. In this work, we solved the FERM structure at a much higher 1.97 Å resolution and we were able to rebuild the segment (363–375) in this area including the RALPSIP motif. The average B factor values for Cα atoms of Lys375 and Val391 located at the N- and C-termini of this unconnected part are 62 Å^2^ and 77 Å^2^, respectively. These values are relatively close to an average B factor value for all protein atoms of the model is (45 Å^2^ - Table 1), which indicates correct model rebuilding for the two segments flanking this unconnected part (residues 376–390). The N-terminal 3 residues of the expression tag remnant for both FERM molecules could not be defined.

**Table 1:** Data collection and refinement statistics for FERM structure: PDB code 6CB0

Data collection	
Wavelength (Å)	0.9787
Resolution range (Å)	123.90–1.97 (2.08–1.97)
Space group	P 21 21 21
Unit cell	50.15123.90135.16 90 90 90
Total reflections	400,847
Unique reflections	55,766 (6816)
Multiplicity	7.2 (7.2)
Completeness (%)	92.43 (78.50)
Mean I/sigma (I)	21.5 (1.99)
Wilson B-factor	35.34
R-merge (%)	5.2 (82.3)
Refinement	
Resolution range (Å)	50.01–1.97 (2.02–1.97)
Number Reflections/unique	55,679 / 52,861 (4186)
Completeness (%)	92.43 (99.98)
Mean I/sigma(I)	19.8 (2.0)
Reflection used for Rfree (%)	5.1
R-work (%)	17.3 (24.7)
R-free (%)	21.2 (29.0)
Number of non-hydrogen atoms	5890
Macromolecules	5491
Water	399
Protein residues	700
RMS (bonds)	0.019
RMS (angles)	1.79
Ramachandran favored (%)	97.4
Ramachandran allowed (%)	2.5
Ramachandran outliers (%)	0.1
Clashscore	2.59
Average B-factor	45.35
Macromolecules	45.06
Solvent	49.30

Statistics for the highest-resolution shell are shown in parentheses

The overall conformation of the FERM molecule displays strong structural similarity with other FERM domains of ERM family members ezrin, radixin, and moesin, consisting of three subdomains (labeled F1, F2, and F3, Fig. 1). Optimal superposition of two FERM molecules in the asymmetric unit results in 2149 equivalent atoms with the RMSD values of 0.438 Å (calculated in PyMOL). The major differences between the two molecules are observed in two loops in subdomains F2 and F3, which is very similar to the crystal structure of the search model PDB 2AL6. Also, similar to the search model, there is non-crystallographic symmetry for the FERM dimer in the crystal packing.

### Comparison between our structure at 1.97 Å and previously published structures

**Fig. 2:**
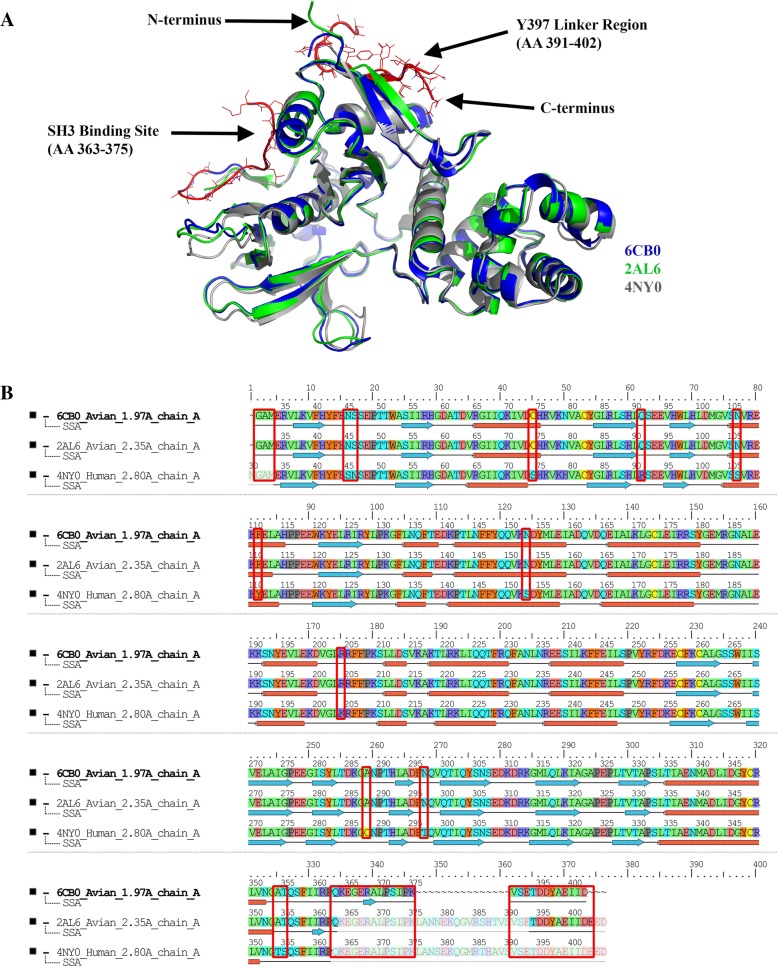
Alignment of our FAK FERM structure with previously published avian and human structures. **a** Structural alignment of our high resolution FERM structure (6CB0 – blue) with published avian (2AL6 – green) and human (4NY0 – grey) FERM structures. Alignment was performed in PyMOL software. Note, key regions in our structure that show unique differences in electron density are highlighted in red. **b** Multiple sequence alignment of FAK FERM structures 6CB0, 2AL6, and 2NY0 performed with the Schrodinger multiple sequence viewer tool. Residue coloring is based on side chain chemistry. Key regions of extra electron density and non-conserved residues between human and avian sequences are boxed in red

Next, we compared our FAK FERM crystal structure (PDB 6CB0) to previously published crystal structures at 2.35 Å (PDB 2AL6) and 2.8 Å (PDB 4NY0). We first started with sequence alignment, structural superposition, and RMSD calculation using PyMOL software (Fig. 2a). Structure 2AL6 aligned with 6CB0 at a RMSD of 0.27 Å and 2050 equivalent atoms. Structure 4NY0 aligned with 6CB0 at a RMSD of 0.709 Å and 2143 equivalent atoms. Overall, all three FERM structures are quite similar other than key differences in the Y397 FERM-Kinase linker region (residues 391–402) and the Src SH3 binding site (residues 363–375). Our structure (6CB0) has unique density from residues 363–375 not present in other structures and shows a different conformation at residues 391–402. The average B factor for all atoms within region 363–375 and 391–402 is 54.6 Å^2^ and 59.0 Å^2^, respectively. Also, there are minor differences in loop regions within the F3 lobe of all three FERM domains. Multiple sequence alignment of the three FERM domain proteins showed a high degree of sequence identity (96%) and homology (99%) between human and avian sequences (Fig. 2b). Intriguingly, regions 391–402 and 363–375 with major differences in electron density share 100% sequence identity.

### Differences in region surrounding FAK Y397 and F1 lobe

**Fig. 3:**
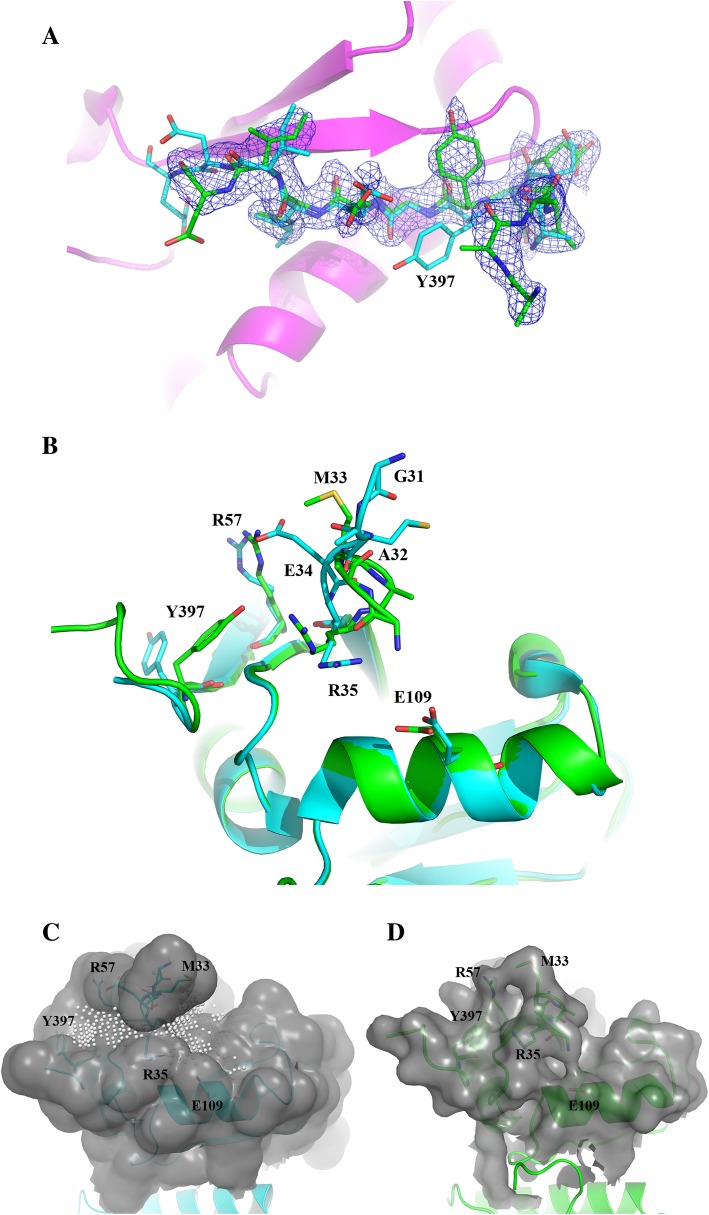
Difference in Y397 region between our structure and previously published FERM structure. **a** Comparison of Y397 within FERM-kinase linker region between our structure (green) and PDB 2AL6 (cyan). 2Fo-Fc electron density map contoured at 1.0 σ is shown in mesh. Note, common ribbon structure between both proteins is shown in magenta. **b** Comparison of FERM F1 lobe residues nearby Y397 in our structure (green) and PDB 2AL6 (cyan). **c** Depiction of druggable pocket nearby Y397 in PDB 2AL6. Note, SiteScore = 0.9. **d** Depiction of region nearby Y397 in our FERM structure. Note, there is an absence of a discernible pocket and SiteMap did not identify this region as druggable

Upon further examination in PyMOL we noticed a significant difference in electron density of key activation residue Y397 and surrounding residues in the FERM-kinase linker as well as residues in the F1 lobe proximal to Y397. In our structure, Y397 is rotated inwards toward the protein and occupies a pocket formed by R35, R57, and H58, however in PDB 2AL6, residue Y397 is pointed outward towards the solvent (Fig. 3a). Additionally, there are alternative conformations of residues G31, A32, M33, E34, R35, R57, and E109 which cause the disappearance of a pocket formed by these residues in PDB 2AL6 that has been previously exploited by computational drug discovery efforts (Fig. 3b). Furthermore, our structure shows unique differences in this area not previously observed in both chains of PDB 2AL6.

To calculate the potential druggability differences between our structure and PDB 2AL6, we subsequently utilized the Schrodinger SiteMap tool, which uses a grid-based searching algorithm around the solvent-exposed surface of the protein to predict enclosed regions that favor small molecule binding. SiteMap analysis of the Y397 region in PDB 2AL6 revealed a defined pocket with a SiteScore of 0.90 (a SiteScore > 0.8 indicates a druggable site) whereas similar analysis of our 1.97 Å structure resulted in the inability to predict any druggable site near Y397 (Fig. 3c and d). These results indicate a significant difference in computationally-predicted druggability of the Y397 region between the two FAK FERM structures.

### Novel conformation of the Src SH3 binding motif

**Fig. 4:**
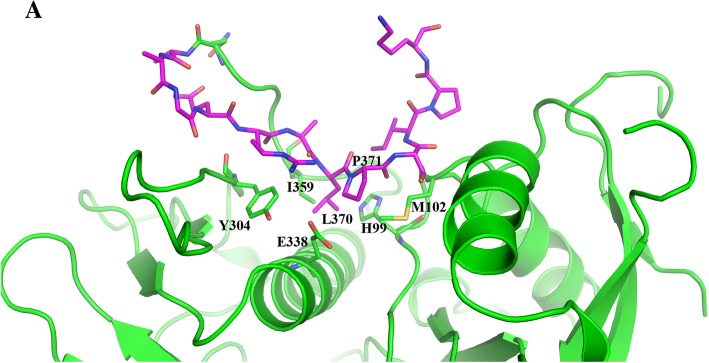
Intramolecular Interactions of Src SH3 Binding Site. **a** Structural representation of the Src SH3 binding site (magenta) forming intramolecular interactions with a cleft in between the FERM F1 and F3 lobes (green). Note, key residues of contact are labeled

The previous crystal structure of the FAK FERM domain (4NY0) did not have adequate electron density for the critical Src SH3 binding motif (residues 363–375) to be visible. In our structure we have better resolution of this region (^363^QKEGERALPSIPK^375^) and therefore have found a previously unidentified conformation of this area. As shown in Fig. 4, residues L370 and P371 of the SH3 binding motif from FERM molecule A pack into a pocket formed by residues I359, Y304, E338, M102, and H99 in between the F1 and F3 lobes of the protein. Residues 372–375 of the motif bind along the side of the F1 lobe however visibility of residues 376–390 cannot be determined. FERM molecule B has a different conformation more similar to previously published FERM structure (4NY0). Altogether, these data suggest an intramolecular interaction between the Src SH3 binding site and the FERM domain which may regulate the activity of FAK and downstream signaling.

## Discussion

Several reports have been published focusing on the discovery of FAK inhibitors that directly target the FERM domain proximal to key residue Y397 as opposed to the ATP-binding pocket of the kinase domain [21–24]. These studies have all utilized computer-aided drug discovery methodology based on the previously published crystal structure of the avian FAK FERM domain (PDB 2AL6) and a putative pocket on the FERM F1 lobe nearby Y397. Here, we report a higher resolution x-ray crystal structure of the FAK FERM domain and reveal novel structural information of the protein that has implications on the druggability of FAK FERM nearby Y397. We identified an alternative conformation of Y397 and the residues encompassing a nearby pocket on the F1 lobe in our structure compared to the previously published structure (PDB 2AL6). Computational prediction of this pocket in our structure suggested that the Y397 region has poor druggable characteristics, contrasting previous reports [21–24]. We speculate that the Y397 region, which is part of the FERM-kinase linker and proximal to the F1 lobe, is highly dynamic in vivo and may represent a combination of conformations observed in our structure as well as previous structures. Furthermore, it is uncertain whether the Y397 region identified here and in other structures represents the biologically relevant conformation or a result of crystal packing. Further structural confirmation of the Y397 region by solution-based studies such as NMR is necessary to fully understand the structure and function of this critical region.

It is important to note that although previous FERM Y397 drug discovery efforts have identified candidate molecules with interesting cell-based data [21–24], there are no inhibitors to date with valid structural evidence of binding specificity (i.e. x-ray co-crystal structures, NMR HSQC chemical shift perturbation studies, etc.). In these studies, micromolar concentrations of compound were required for cellular effects on viability/apoptosis and on-target FAK inhibition was only measured by western blotting and in vitro kinase assays. Only one study [24] performed a biophysical assay (Bio-Layer Interferometry), using high concentrations of compound (500uM) and no report of binding affinity (K_D_). An explanation for the disconnect between biological activity and structural validation of FAK FERM inhibitors could be that FAK FERM x-ray crystallography studies do not correctly sample the pharmacologically-active conformation required for small molecule binding. In addition, putative FAK FERM inhibitors may be indirectly acting on the FAK pathway through direct binding of an upstream molecule such as a transmembrane receptor. As such, our data bring concerns to the druggability of the Y397 region and suggest that structural confirmation of binding and alternative drug discovery/structural biology strategies (i.e. fragment screening, NMR, covalent tethering) rather than a sole computational approach based on x-ray should be employed to find small molecule inhibitors of the Y397 region.

Another important finding of our FAK FERM structure was the visibility of the Src SH3 binding site (^363^QKEGERALPSIPK^375^), which was not found in previous FAK FERM structures [25, 26]. In our structure, the Src SH3 binding site makes intramolecular contacts with a pocket in between the FERM F1 and F3 lobes. This finding may represent a novel allosteric form of regulation of FAK activation through modulation of the Src-FAK interaction. We speculate that the intramolecular interactions between the Src SH3 binding site and the FERM F1/F3 lobes may be an “autoinhibited” conformation that prevents binding of Src to FAK and therefore the full activation of FAK by phosphorylation of Y576, Y577, Y861, and Y925. Additionally, our structure implicates residues between the F1 and F3 lobes (I359, N339, M102, and H99) as potential sites of allosteric modulation as a means to relieve SH3 binding site-F1/F3 autoinhibitory interactions and allow Src binding.

## Conclusions

In all, we present a higher resolution crystal structure of the FAK FERM domain that reveals novel information on the druggability of the Y397 FERM region and the intramolecular interactions of the Src SH3 binding site. These data may guide future drug discovery efforts in the development of FAK inhibitors that directly target the region proximal to key residue Y397. Also, this new crystal structure may serve to support future hypotheses investigating the structural regulation of FAK through modulation of the Src SH3 binding site.

## Methods

### Protein expression and purification

Avian FERM domain protein (residues 31–405) was expressed and purified similarly as previously reported [25]. The pET-chFERM construct was kindly provided by Dr. Michael J. Eck. In brief, BL21 (DE3) *E. coli* were transformed and subsequently cultured in LB media until an O.D. at 600 nm of 0.8 was reached. Protein expression was induced by addition of 0.2 mM IPTG and grown in a shaking incubator for an additional 16 h at 30 °C. Cells were then pelleted and lysed with lysis buffer (20 mM Tris-Cl pH 8.0, 200 mM NaCl, 5 mM BME, 10 mM imidazole, 1X Halt Protease Inhibitor Cocktail). Lysate was subjected to one freeze-thaw cycle followed by sonication for 2 min on ice. Supernatant was collected after centrifugation at 12,000 rpm for 15 min and incubated with 3 mL Ni-NTA resin (Thermo) overnight. Resin was then washed with lysis buffer containing 25 mM imidazole and protein was eluted with lysis buffer containing 200 mM imidazole.

The His-tag from the purified FERM-domain sample was removed by AcTEV-protease according to the manufacture protocol (Invitrogen). Briefly, 25 units of AcTEV-protease sample was enough to complete the cleavage of the His-tag from 1 mg of the protein during overnight incubation at 40 C. The cleaved product was removed by filtration of the sample through Ni-NTA Superflow cartridge (5 ml, Qiagen) pre-equilibrated in 20 mM Tris-HCL, pH 8.0, 100 mM NaCl, 1 mM DTT and 20 mM imidazole. The filtrated sample was loaded on a Q-Sepharose column (GE Healthcare) equilibrated in the same buffer except the imidazole and the protein was eluted using a linear gradient of NaCl from 0.1 to 1 M. The fractions of the His-off protein were pooled and applied on a Superdex 200 column (GE Healthcare) for further protein purification. The FERM–domain sample eluted from the gel filtration column was 95% pure as assessed by SDS-PAGE analysis and used for the crystallization experiments.

### Crystallization, data collection and structure determination

Sitting drops in 96-round bottom well crystallization plates (Greiner Bio-One, GmbH, Germany) were set up using a Mosquito Robotic System (TTP LabTech, Hertfordshire, U.K.). Avian FAK FERM domain (residues 31–405) protein sample at concentration of 10 mg/ml in 10 mM Tris-HCl buffer, pH 8.0, 50 mM NaCl and 1 mM DTT was used for the crystallization experiments.

Diffraction quality FERM protein crystals were obtained at 291 K from drops containing 0.5 μl of the protein sample and 0.5 μl of reservoir solution (0.2 M K/Na tartrate, 20% PEG 3350). Diffraction data were collected at 100 K from a single, flash frozen, crystal [28], cryoprotected in 20% (v/v) glycerol, on a Mar CCD-300 detector at LS-CAT (Advanced Photon Source, Argonne National Laboratory). Diffraction data were then indexed and processed with XDS software [29]. The crystal belonged to orthorhombic space group P212121 and contained two FERM molecules per asymmetric unit. The structure of FERM-domain was solved by molecular replacement using Phaser software [30] with the structure of avian FERM (residues 31–405) (PDB code 2AL6) as a search model. The final model of FERM-domain was progressively refined by performing several cycles of manual model building using COOT [31], subsequently followed by structure refinement with REFMAC [32]. Local NCS restraints were not used because of initial high resolution data. Crystallographic data and refinement statistics (including Ramachandran statistics) are shown in Table 1. Adequate side chain electron density was not found for residues E34, E93, E118, K141, K216, K218, K310, D311, R312, K313, E325, Q356, K364, E365, V391, S392, E393, and therefore they are not present in the final structure. Coordinates have been deposited in the Protein Data Bank with accession number 6CB0.

### In silico modeling

The PyMOL molecular graphics program (Schrödinger, Inc.) was utilized for general structural representation of the FERM domain and publication-quality images were made using the ray-trace command. PyMOL was also used for sequence alignment and structural superposition using the align command. Multiple sequence alignment was performed using the Schrödinger Multiple Sequence Viewer application. For druggability prediction analysis of our crystal structure compared to previously published structures, we used the program SiteMap (Schrödinger, Inc.) as described [33]. SiteMap uses a grid-based searching algorithm, similar to the Goodford GRID algorithm [34]. We utilized the following settings: report up to 10 binding sites, a more restrictive definition of hydrophobicity, standard grid, and lower constraints to detect shallow binding sites. Identified binding sites with a calculated SiteScore > 0.8 were classified as “druggable” sites having a high probability for sub-micromolar ligand binding.

## Abbreviations

FAK: Focal Adhesion Kinase
FERM: Band 4.1, Ezrin, Radixin, Moesin
PDB: Protein Data Bank
SH3: Src Homology 3
